# Vertically Free-Standing Ordered Pb(Zr_0.52_Ti_0.48_)O_3_ Nanocup Arrays by Template-Assisted Ion Beam Etching

**DOI:** 10.1186/s11671-016-1369-x

**Published:** 2016-04-27

**Authors:** Xiaoyan Zhang, Dan Tang, Kangrong Huang, Die Hu, Fengyuan Zhang, Xingsen Gao, Xubing Lu, Guofu Zhou, Zhang Zhang, Junming Liu

**Affiliations:** Institute for Advanced Materials and Guangdong Provincial Key Laboratory of Quantum Engineering and Quantum Materials, South China Academy of Advanced Optoelectronics, South China Normal University, Guangzhou, 510006 China; Institute of Electronic Paper Displays, South China Academy of Advanced Optoelectronics, South China Normal University, Guangzhou, Guangdong Province 510006 China; Academy of Shenzhen Guohua Optoelectronics, Shenzhen, 518110 China; Laboratory of Solid State Microstructures and Innovation Center of Advanced Microstructures, Nanjing University, Nanjing, 210093 China

**Keywords:** Vertically free-standing, PZT nanocups, AAO, Ion beam etching

## Abstract

In this report, vertically free-standing lead zirconate titanate Pb(Zr_0.52_Ti_0.48_)O_3_ (PZT) nanocup arrays with good ordering and high density (1.3 × 10^10^ cm^−2^) were demonstrated. By a template-assisted ion beam etching (IBE) strategy, the PZT formed in the pore-through anodic aluminum oxide (AAO) membrane on the Pt/Si substrate was with a cup-like nanostructure. The mean diameter and height of the PZT nanocups (NCs) was about 80 and 100 nm, respectively, and the wall thickness of NCs was about 20 nm with a hole depth of about 80 nm. Uppermost, the nanocup structure with low aspect ratio realized vertically free-standing arrays when losing the mechanical support from templates, avoiding the collapse or bundling when compared to the typical nanotube arrays. X-ray diffraction (XRD) and Raman spectrum revealed that the as-prepared PZT NCs were in a perovskite phase. By the vertical piezoresponse force microscopy (VPFM) measurements, the vertically free-standing ordered ferroelectric PZT NCs showed well-defined ring-like piezoresponse phase and hysteresis loops, which indicated that the high-density PZT nanocup arrays could have potential applications in ultra-high non-volatile ferroelectric memories (NV-FRAM) or other nanoelectronic devices.

## Background

In recent years, increasing efforts have been made to synthesize and understand ferroelectric nanostructures because of their peculiar physical properties, such as their finite size effects and unusual phase transitions [1, 2], offering a wide range of potential applications in nanoscale piezoelectric actuators [3], force and acceleration sensors [4, 5], ultrasonic transducers [6], and non-volatile ferroelectric random access memory (NV-FRAM) devices [7]. Due to the current trends of high integration and miniature in semiconductor industry, ferroelectric memories have been receiving more and more attention due to their unique advantages such as high density, low power consumption, and high read/write speed [8–10].

Lead zirconium titanate Pb(Zr_0.52_Ti_0.48_)O_3_ (PZT), a solid solution of the perovskites lead zirconate and lead titanate, is a prominent ferroelectric material that has stimulated tremendous fundamental and applied researches due to its high spontaneous polarization abilities, piezoelectric coefficient, dielectric permittivity, and pyroelectricity [11–13]. Applications of PZT nanostructures include tunable photonic crystals, ferroelectric random access memory (FRAMs), terahertz emission, fluidic delivery, and nanosensors [14–16]. In addition, the significant need of miniaturization of electronic devices leads to more extensive usage of PZT FRAMs based on the low-dimensional nanostructures [17]. Sol-gel process is one of the promising routes among many suitable methods for the preparation of nanostructured PZT materials, as it leads to products with high chemical homogeneity and purity at comparably low temperatures [18]. However, the synthesis speed of ferroelectric nanotubes (NTs) has been relatively slower than it has been for other materials, which might due to the difficulties associated with the structural and stoichiometric complexity [19]. For large-scale memory device, high-density ordered ferroelectric NTs with a vertically aligned integration on the substrate is one essential. In recent years, high-density vertically aligned ordered ferroelectric NTs can be fabricated using template-assisted method [18]. In particular, anodic aluminum oxide (AAO) templates are with advantages of good ordering, large-area fabrication, paralleled pore arrangement, and tunable size [20]. Nevertheless, the clamping effect degrades the properties with the remaining template around the nanostructure [21, 22]. After getting rid of the template, however, the loss of the mechanical support from AAO and capillary force always resulted in the degradation of the ordered array alignment on the substrate [23, 24]. Obviously, the agglomeration of the nanostructures would greatly affect the device performance. And the agglomeration was mainly due to the high surface tension, which was closely related to the compressive stress of the nanostructures. In order to reduce surface tension, the high-density nanostructures should be preferentially designed with low aspect ratio of height to diameter [25]. Up to now, there have been few reports addressing both the vertically free-standing ordered nanostructure arrays and the structure-property relations of the ferroelectric nanostructures.

In this work, we have successfully developed high-density and well-ordered vertically free-standing (VFS) PZT nanocup arrays on a conductive Pt/Si substrate, by a template-assisted ion beam etching (IBE) method. The low aspect ratio of height to diameter being critical to the vertically free-standing feature of PZT nanocups (NCs) could be well controlled by the ultrathin AAO templates and IBE process. X-ray diffraction (XRD) and Raman spectrum revealed that the as-prepared PZT NCs were in a perovskite phase. By the vertical piezoresponse force microscopy (VPFM) measurements, the well-ordered ferroelectric PZT nanocup arrays showed well-defined ring-like piezoresponse phase and hysteresis loops, which indicated that the nanostructure could have potential applications in ultra-high NV-FRAMs or other oxide nanoelectronic devices.

## Methods

### AAO Fabrication

AAO templates are fabricated by a standard two-step anodization method. Firstly, high-purity aluminum foils (99.999 %, Good fellow Cambridge Limited) were degreased and then annealed at 450 °C for 3 h under argon atmosphere. Then, the Al foils are electrochemically polished in a solution of mixed ethanol and HClO_4_ (3:1 by volume) at 20 V for 5 min to form a mirror-like surface smoothness. The first anodic oxidation process is performed in a 0.3-M oxalic acid with a constant anodic voltage of 40 V. After 24 h, the first alumina layer is removed by a mixed aqueous solution of 6 % H_3_PO_4_ and 1.8 % H_2_CrO_4_ at 45 °C for 11 h. The second anodic oxidation process is performed under the same conditions as the first one, with a 300-s oxidation time to grow the AAO membrane with a thickness of about 200 nm. A 5 wt.% phosphoric acid was used to widen the pore diameter to about 80 nm. In order to realize the AAO membrane bonded onto Pt/Si substrate, a polymer-assisted method was used. First, a thin layer of polystyrene (PS) (1 wt.% PS/CHCl_3_ solution) was spin-coated onto the top of AAO film, followed by a 90 °C solidification heating. After that, a CuCl_2_ solution (6.8 g CuCl_2_ + 100 ml 37 % HCl + 200 ml distilled water) was used to remove the Al foil on the back side of the AAO. The membrane was then immersed in 5 wt.% phosphoric acid at 35 °C for 30 min, rinsed with distilled water, and then dried under ambient conditions to removal barrier layer [26]. The whole PS/AAO membrane still floating on the surface of deionized water was transferred to a desired substrate. Subsequently, the van der Waals forces at the interface exhibit an excellent bonding which can still survive even under strong mechanical evacuation. The PS can be completely removed by a pyrolysis process.

### Template-Assisted IBE Process

A 0.3-M PZT sol-gel precursor with the molar ratio of 1:0.52:0.48 was prepared by dissolving zirconium propoxide (Zr(CH_2_CH_2_CH_3_O)_4_) and isopropyl titanate (Ti(C_4_H_9_O)_4_) into 2-methoxyethanol (C_3_H_8_O_2_), with acetic acid and propanol added as the solvents, stirring until completely dissolved. Then, 10 % excess lead acetate trihydrate (Pb(CH_3_CO_2_)_2_·3H_2_O) was added with the purpose of compensating the lead loss and preventing forming the second phase of pyrochlore-type in annealing process [27]. The precursor solution was stirred for 24 h at room temperature and then aged for 1 week. The PZT sol was dipped into the AAO template for nearly 10 h at the room temperature for atmosphere pressure. After infiltration, excess PZT accumulated on the surface is swept with nitrogen gas gun. The samples were then annealed at 700 °C for 20 min in air by a rapid thermal process (RTP) to experience a crystallization process. Afterwards, in an ambient pressure of 5.6 × 10^−4^ mbar at room temperature, the specimen was etched by Ar IBE (MIBE-150C). The first 10-min IBE was used to remove off the residual PZT film that covered on the surface of AAO template after the annealing process. The continuous IBE reduced both the thicknesses of the AAO and inside PZT. The total etching time was about 20 min with a vertical incident ion beam to the specimen. The etching energy was set to a cathode current of 11.5A, anode voltage of 55 V, plate voltage of 300 V, ion accelerating voltage of 250 V, and neutralization current of 13A and bias current of 1.2A. After the IBE process, the remaining AAO was completely removed off by dipping the specimen into sodium hydroxide solution.

### Characterizations

The morphologies of nanostructure were carried out ex situ with a field-emission scanning electron microscope (FESEM ZEISS Ultra 55). The crystalline structural characterizations were investigated by XRD using a Philips X’Pert Pro Cu Kα diffractometer with an x-ray wavelength of 0.15406 nm. The Raman spectra were measured at room temperature by the Renishaw inVia-Reflex Raman microscope with an excitation wavelength of 633 nm. The laser power is set to 10 % of the maximum. The ferroelectric nanostructures were measured by a piezoresponse force microscopy (PFM) (Cypher, Asylum Research).

## Results and Discussion

### PZT Nanocup Arrays by Template-Assisted IBE

**Fig. 1:**
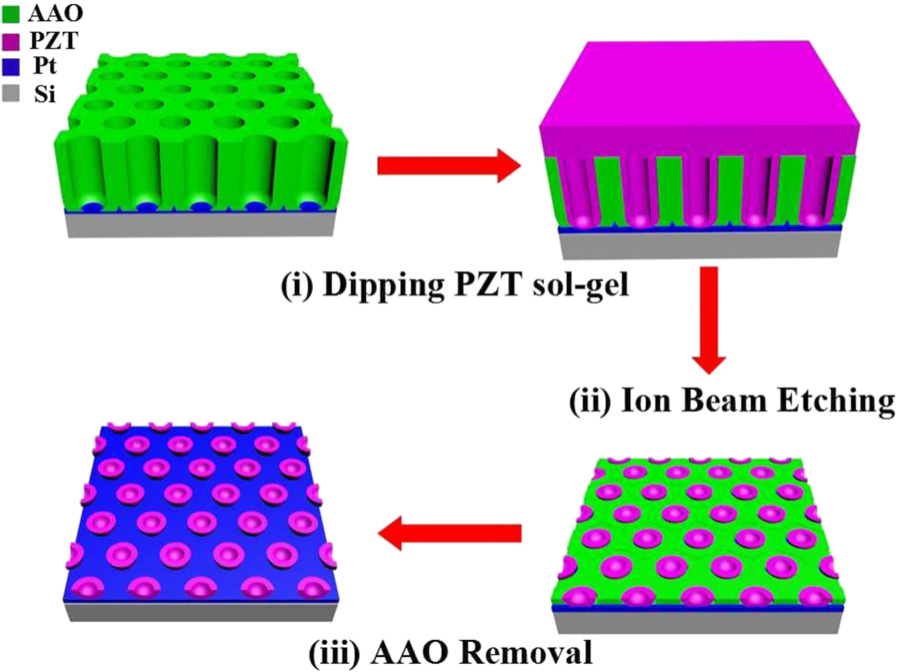
Schematic diagram of the three fabrication procedures for the ordered VFS PZT NC arrays. **i** Dip PZT sol-gel into the AAO template. **ii** Remove the redundant PZT at the top surface of AAO by IBE. **iii** Remove the remaining AAO template to form the ordered VFS PZT NC arrays on Pt/Si substrate

The template-assisted atmosphere pressure infiltration process for PZT nanocup arrays was schematically illustrated in Fig. 1. The synthesis process shown here mainly consists of three following steps. Firstly, the pore-through AAO template was dipped into the prepared PZT sol-gel precursor (detailed in Methods Section). And then, the samples were annealed in RTP to form the crystallization PZT nanostructure. By the reported template-assisted sol-gel method [28], the presence of PZT film on the top surface of AAO would prevent the selectively chemical etching of AAO, since the AAO was completely covered by the PZT film. Therefore, the IBE pretreatment was used to get rid of the top surface PZT film, which would effectively bare the top surface of AAO to the chemical etching solution. Secondly, the IBE was adopted to remove the redundant PZT located at the surface of the AAO template. Meanwhile, the heights of tubular wall of the AAO and the implemented PZT were both reduced. Finally, after the AAO template being completely removed off by selectively chemical etching, the ordered VFS PZT nanocup arrays were remained on the substrate.

**Fig. 2:**
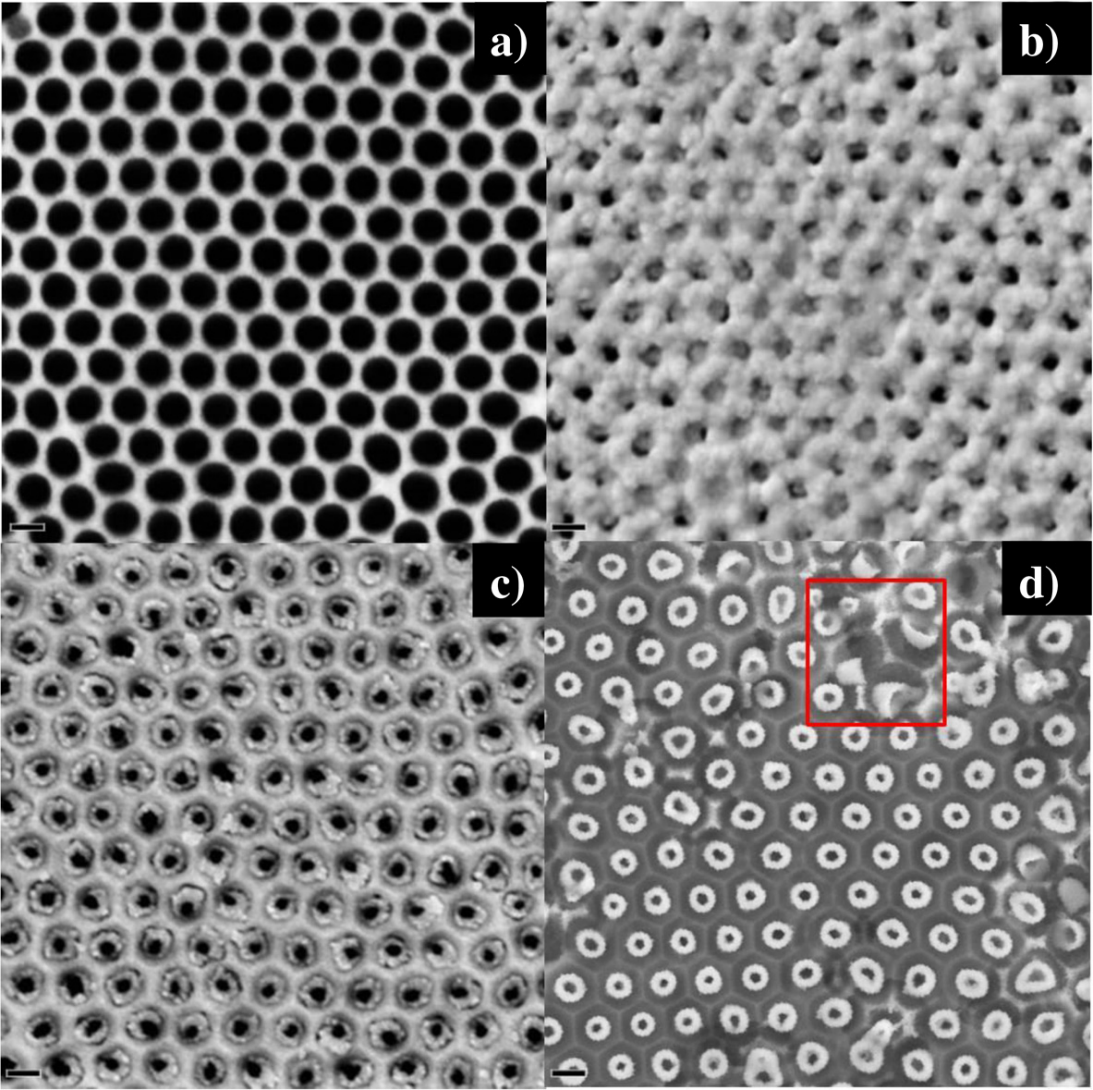
Top view SEM images of the fabrication process of VFS PZT NC arrays. **a** The pore-through AAO template with an enlarged pore diameter of about 80 nm. **b** AAO template with PZT sol-gel infiltration after the annealing process. **c** AAO template with embedded PZT nanostructure after etching off the PZT top layer on the top surface of AAO by an IBE. **d** VFS PZT NC arrays on Pt/Si substrate after selectively chemical etching off AAO. The scale bar is 100 nm

The top view SEM images of the corresponding fabrication procedures (illustrated in Fig. 1) were presented in Fig. 2. Before the AAO was transferred to the platinized silicon substrate, the AAO was pretreated in the 5 wt.% phosphoric acid at 35 °C for 35 min, rinsed with distilled water, and then dried under ambient conditions, in order to get rid of the barrier layer at the bottom side. Meanwhile, such a treatment to AAO introduced pore widening as well, and the increase of pore diameter from 40 to 60 nm can be recognized in Fig. 2a. The pore-through AAO template is also essential to keep the contact well between the conductive platinum substrate and the embedded PZT nanostructure to realize the lateral piezoelectric testing. After the atmosphere pressure infiltration process of PZT sol-gel precursor, the sample was annealed in atmosphere by a RTP. Afterwards, a homogeneously porous PZT layer accumulated at the top surface of AAO (as shown in Fig. 2b), which was an inevitable by-product using the atmosphere pressure infiltration template-assisted sol-gel method [28]. And it should be realized that such a by-product will greatly hinder the selectively chemical etching of the AAO. In this report, we brought in a successive IBE process to get rid of the PZT layer to expose the underlying AAO and embedded PZT nanostructure. During the IBE, the neutral-charged Ar ion beam transmitted its kinetic energy to the atoms of the specimen to sputter out. The high-energized Ar ion beam can also play as a dustman to clean the sputtering redeposition on surfaces to speed up the etching process [29]. Due to the bombarding effect of Ar ion beam, the PZT layer on the top surface of AAO was first removed off. Successively, with the ongoing of IBE, the tubular nanostructure of AAO and the embedded PZT nanostructure were both etched to be thinner. With a proper duration of IBE, the porous PZT layer on the top surface of AAO was etched off completely, and the ring-like PZT nanostructure arrays could be distinguished to be embedded in each AAO pore by the top view SEM image (Fig. 2c). After selective etching off AAO, the VFS PZT nanocup arrays were remained on the Pt/Si substrate (Fig. 2d). The mean diameter of the PZT NCs was about 80 nm, with a wall thickness of about 20 nm. And the distance between the adjacent NCs was about 100 nm, being the same with the interpore distance of AAO, resulting in a higher density of PZT nanostructure arrays about 1.3 × 10^10^ cm^−2^ compared to the reported arrays [28, 30]. Meanwhile, the cup-like nanostructure of PZT with a small height to diameter ratio was also confirmed by a few collapsed PZT NCs on the substrate (as shown in the selected red square box of Fig. 2d).

### Phase Analysis

**Fig. 3:**
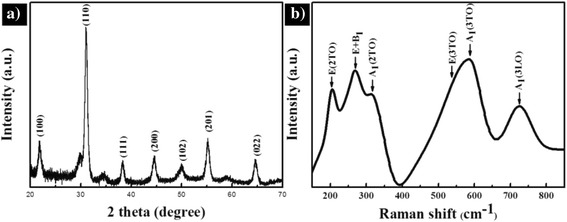
X-ray diffraction pattern and Raman spectrum of the ordered VFS PZT NC arrays measured at room temperature. **a** XRD peak pattern of annealed PZT NC arrays. **b** The corresponding Raman spectrum

XRD was used to investigate the phase and crystallinity of the PZT NCs. In order to get strong signals, the high-density VFS PZT NC arrays were characterized. Generally, the as-deposited PZT film was amorphous, and a post deposition annealing was required to transform the film from the amorphous to the desirable ferroelectric perovskite phase. The amorphous structure will first transform into an intermediate pyrochlore phase, and then, the pyrochlore phase will transform into the perovskite phase at an annealing temperature higher than 650 °C [27]. The perovskite phase grew from the surface of the pyrochlore film. To avoid the pyrochlore phase, the annealing temperature was set to 700 °C and 10 % excess lead acetate trihydrate (Pb(CH_3_CO_2_)_2_·3H_2_O) was added in our experiments. Figure 3a is a typical XRD peak pattern of the PZT nanocup arrays, with an annealing at 700 °C. The diffraction peak at 2*θ* = 31.35°, corresponding to the PZT (110) plane, was obviously stronger than the other peaks. The strong and sharp diffraction peaks are coincident with the peak pattern of the PZT perovskite crystalline structure [31, 32]. In addition to peaks belonging to the perovskite phase, small Bragg reflections of a pyrochlore phase were also observed in the XRD peak pattern. The formation of an intermediate pyrochlore or fluorite phase was kinetically favored over the perovskite phase, during synthesis of lead-based ferroelectric films on substrates and free-standing PZT films, respectively [27, 33]. Therefore, the existence of a surface pyrochlore phase cannot be precluded.

To further characterize the composition, the Raman spectrum of PZT nanocup arrays was shown in Fig. 3b. Since the PZT tetragonal space group belongs to the P4mm, the typical 3A1 + B1 + 4E mode in Raman peaks can be clearly identified, with six main peaks: 205, 275, 317, 533, 593, and 724 cm^−1^, corresponding to the E(2TO), E(silent) + B1, A_1_(2TO), E(3TO), A_1_(3TO), and A_1_(3LO) modes, respectively. With the six phonon modes found in the Raman spectrum, which is the typical one of PZT perovskite phase, it was further confirmed that the as-prepared PZT had been in its perovskite phase [3].

### Ferroelectric Properties

**Fig. 4:**
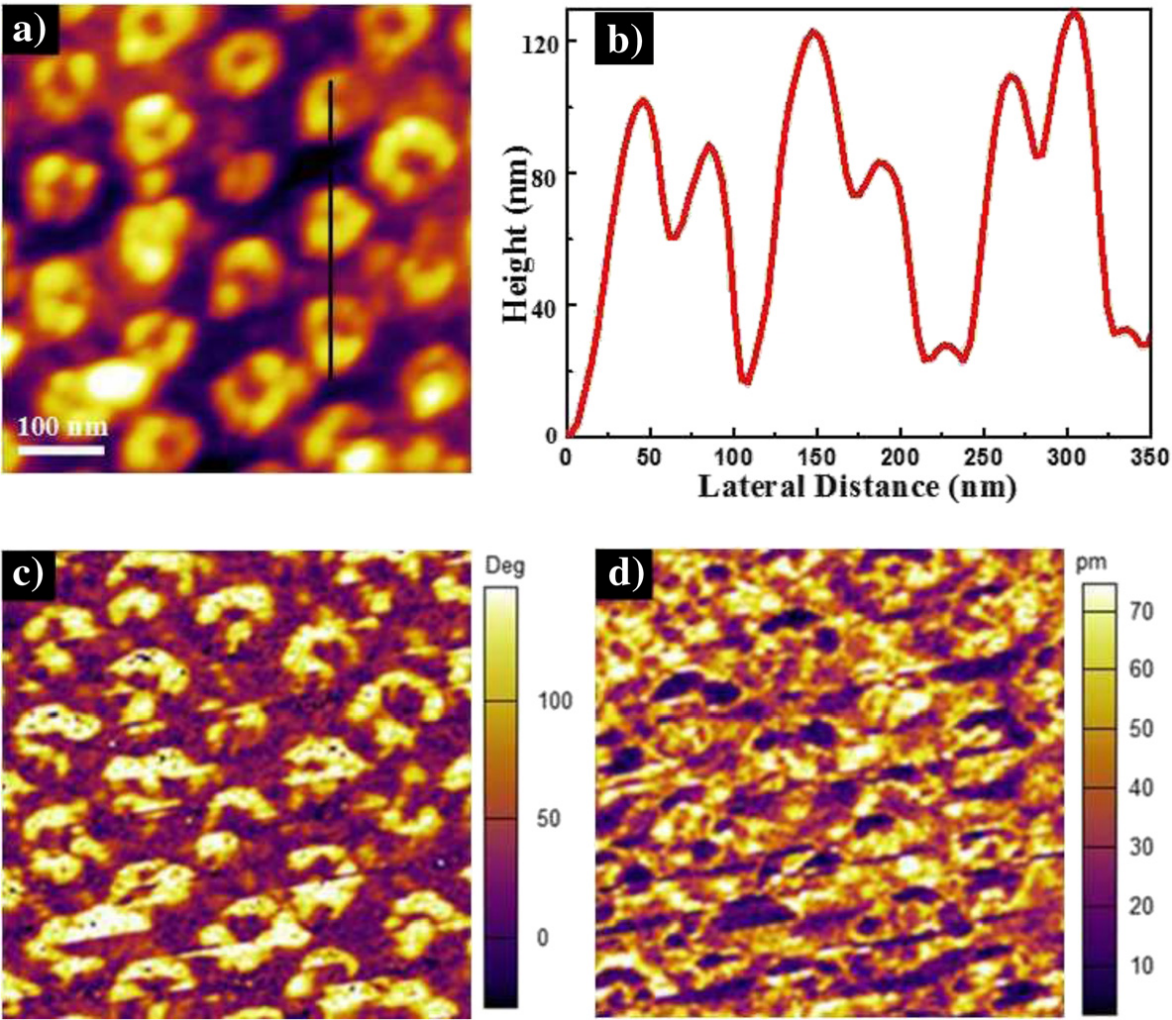
Piezoelectric response of the ordered VFS PZT NC arrays on Pt/Si substrate. **a** Topologically image. **b** Height data for three PZT NCs along the *black line* marked in **a**. The corresponding **c** phase image and **d** amplitude image

PFM can provide non-destructive high spatial resolution and uniform localization electric field at the junction between the conductive tip and ferroelectric surface, obtaining both the domain structures and the electrical properties of the nanometer scale ferroelectric structures [34]. To characterize the ferroelectric properties of the well-ordered VFS PZT nanocup arrays, VPFM measurements were performed and demonstrated in Fig. 4, including the atomic force microscopy (AFM) topography, cross-sectional height data, phase micrograph, and the piezoresponse amplitude of the same selected zone of PZT nanocup arrays. The surface topology (as shown in Fig. 4a) exhibited a uniform and well-aligned ordering. And the diameter and wall thickness of the PZT NCs was about 80 and 20 nm, respectively, which was consistent with the observation of the SEM images. In Fig. 4b, from the height data of three linear NCs (marked in Fig. 4a), we concluded that the average height of PZT NCs was about 100 nm. The dark and bright areas in the phase micrograph (Fig. 4c) correspond to the up-polarization and down-polarization state, respectively, indicating the well-defined piezoresponse of the ordered PZT nanocup arrays. The contrasts in amplitude piezoresponse (Fig. 4d) represent the magnitudes of the piezoelectric signals, which are much higher on the NCs.

**Fig. 5:**
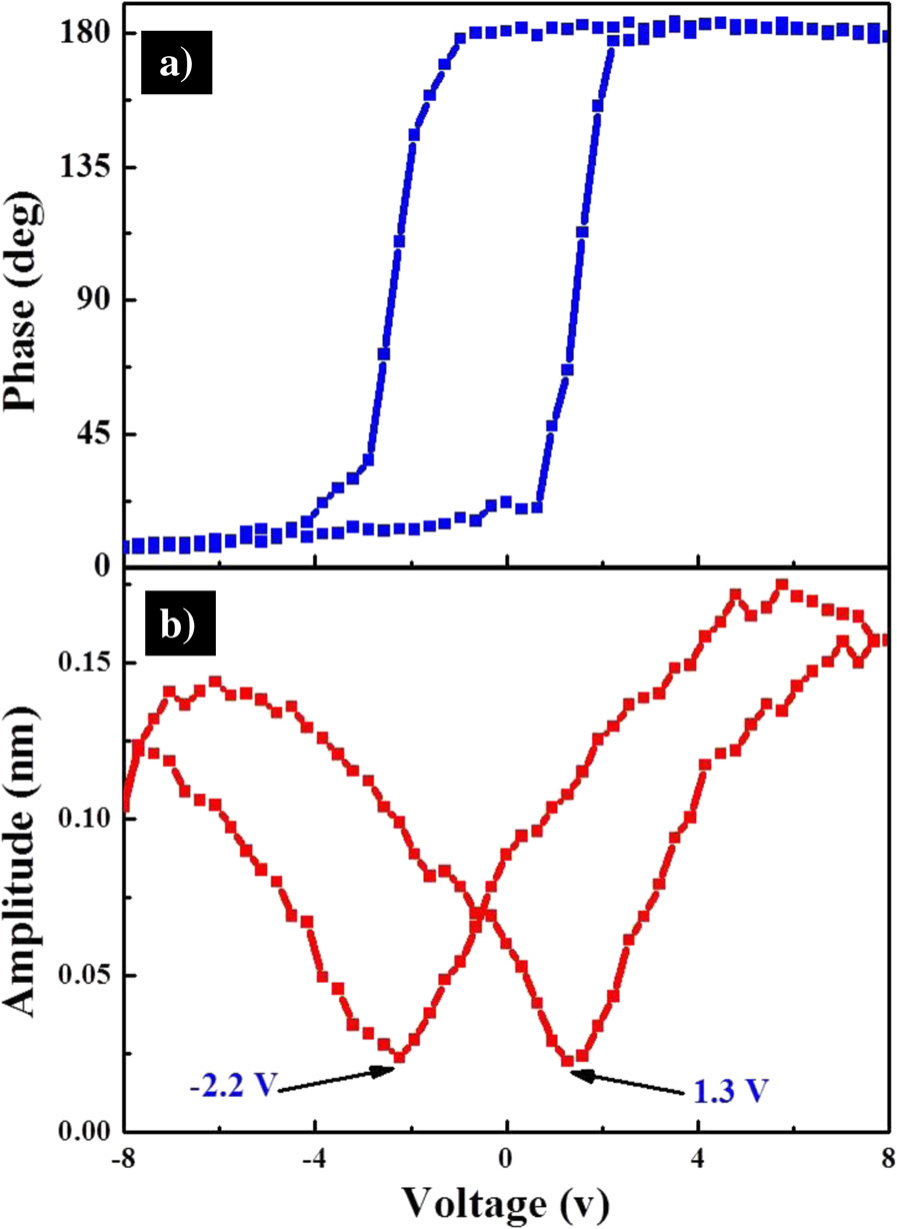
Local piezoresponse hysteresis loops acquired on a single VFS PZT NCs. **a** Phase-voltage piezoresponse hysteresis loops. **b** Amplitude-voltage loop

Furthermore, to examine the local ferroelectric properties, the piezoelectric hysteresis loops on a single PZT nanocup were measured. The square-shaped phase-voltage piezoresponse hysteresis loop and the butterfly-like amplitude-voltage loop were present in Fig. 5a, b, respectively. With the bias voltage increasing from −8 to +8 V, the phase change was about 175° plotted in Fig. 5a, being slightly smaller than 180°. Generally, such a phase change indicated that the low aspect ratio of nanostructure had resulted in an easier polarization switching [31]. Meanwhile, a well-developed butterfly-shaped amplitude loop can be observed in Fig. 5b. The coercive fields of the PZT nanocup were −2.2 and +1.3 V, indicating that the polarization reversal was asymmetric. Such a phenomenon could be due to the built-in fields from the work-function difference between the top and bottom electrodes. The PFM conductive needlepoint acted as the top electrode was the platinum-iridium, while the bottom electrode was the platinum-silicon. Normally, the platinum and the iridium have their work functions of around 5.65 and 5.27 eV, respectively, which produces an overall theoretical built-in voltage of 0.38 eV. This breaks the equivalence of two polarization states and provides a strong tendency to alight the domains to a preferred orientation [35–37]. Additionally, the surface charges stored at the interface between PZT and Pt electrode may contribute to the observed asymmetric polarization states [38]. Therefore, the PZT nanocup arrays with good polarization switching properties could potentially act as a memory element in NV-FRAM devices [39].

## Conclusions

In summary, we have successfully fabricated well-ordered VFS PZT nanocup arrays, by the template-assisted IBE strategy. The PZT nanocup arrays show the same pattern as the AAO template, and the high density of the hexagonal close packed NCs is up to 1.3 × 10^10^ cm^−2^. XRD and Raman spectrum had been performed to demonstrate the perovskite phase of the VFS PZT NCs. The well-defined ring-like piezoresponse phase and amplitude performances of these ordered ferroelectric PZT nanocup arrays were confirmed by VPFM. All experimental results indicated that the ordered VFS PZT nanocup arrays could have potential applications in NV-FRAM devices.

## Abbreviations

AAO: Anodic aluminum oxide
AFM: atomic force microscopy
FESEM: field-emission scanning electron microscope
FRAMs: ferroelectric random access memory
IBE: ion beam etching
NCs: nanocups
NTs: ferroelectric nanotubes
NV-FRAM: nonvolatile ferroelectric random access memory
PS: polystyrene
PZT: lead zirconate titanate Pb(Zr_0.52_Ti_0.48_)O_3_
RTP: rapid thermal process
VFS: vertically free-standing
VPFM: vertically piezoresponse force microscopy
XRD: X-ray diffraction
